# The Global, Regional, and National Burden and Trends of NAFLD in 204 Countries and Territories: An Analysis From Global Burden of Disease 2019

**DOI:** 10.2196/34809

**Published:** 2022-12-12

**Authors:** Huilong Chen, Yuan Zhan, Jinxiang Zhang, Sheng Cheng, Yuhao Zhou, Liyuan Chen, Zhilin Zeng

**Affiliations:** 1 Department and Institute of Infectious Diseases Tongji Hospital of Tongji Medical College Huazhong University of Science and Technology Wuhan China; 2 Department of Respiratory and Critical Care Medicine National Clinical Research Center of Respiratory Disease, Tongji Hospital, Tongji Medical College Huazhong University of Science and Technology Wuhan China; 3 Department of Spine Surgery The Second Hospital affiliated to Guangdong Medical University Guangdong Medical University Zhanjiang China; 4 Department of Respiratory and Critical Care Medicine University-Town Hospital Chongqing Medical University Chongqing China; 5 Department of Obstetrics and Gynecology Wuhan No1 Hospital Wuhan China

**Keywords:** non-alcoholic fatty liver disease, Global Burden of Disease Study 2019, epidemiologic change, diabetes mellitus type 2, stroke, ischemic heart disease, incidence, prevalence, mortality, disability-adjusted life-years

## Abstract

**Background:**

Nonalcoholic fatty liver disease (NAFLD) poses a substantial socioeconomic burden and is becoming the fastest growing driver of chronic liver disease, potentially accompanied by a poor prognosis.

**Objective:**

We aim to elucidate the global and regional epidemiologic changes in NAFLD during the past 30 years and explore the interconnected diseases.

**Methods:**

Data on NAFLD incidence, prevalence, death, and disability-adjusted life-years (DALYs) were extracted from the Global Burden of Disease Study 2019. The age-standardized incident rate (ASIR), age-standardized prevalent rate (ASPR), age-standardized death rate (ASDR), and age-standardized DALYs were calculated to eliminate the confounding effects of age when comparing the epidemiologic changes between different geographical regions. In addition, we also investigated the correlation between the NAFLD burden and the sociodemographic index (SDI). Finally, the associations of the 3 common comorbidities with NAFLD were determined.

**Results:**

Globally, the incidence and prevalence of NAFLD both increased drastically during the past 3 decades (incidence: from 88,180 in 1990 to 172,330 in 2019, prevalence: from 561,370,000 in 1990 to 1,235,700,000 in 2019), mainly affecting young adults who were aged from 15 to 49 years. The ASIR increased slightly from 1.94 per 100,000 population in 1990 to 2.08 per 100,000 population in 2019, while ASPR increased from 12,070 per 100,000 population in 1990 to 15,020 per 100,000 population in 2019. In addition, the number of deaths and DALYs attributable to NAFLD increased significantly as well from 93,760 in 1990 to 168,970 in 2019 and from 2,711,270 in 1990 to 4,417,280 in 2019, respectively. However, the ASDR and age-standardized DALYs presented decreasing trends with values of estimated annual percentage change equaling to –0.67 and –0.82, respectively (ASDR: from 2.39 per 100,000 population in 1990 to 2.09 per 100,000 population in 2019; age-standardized DALYs: from 63.28 per 100,000 population in 1990 to 53.33 per 100,000 population in 2019). Thereinto, the burden of death and DALYs dominated the patients with NAFLD who are older than 50 years. Moreover, SDI appeared to have obvious negative associations with ASPR, ASDR, and age-standardized DALYs among 21 regions and 204 countries, although there is no marked association with ASIR. Finally, we found that the incidence and prevalence of NAFLD were positively related to those of diabetes mellitus type 2, stroke, and ischemic heart disease.

**Conclusions:**

NAFLD is leading to increasingly serious health challenges worldwide. The morbidity presented a clear shift toward the young populations, while the heavier burden of death and DALYs in NAFLD was observed in the aged populations and in regions with relatively low SDI. Comprehensive acquisition of the epidemiologic pattern for NAFLD and the identification of high-risk comorbidities may help policy makers and clinical physicians develop cost-effective prevention and control strategies, especially in countries with a high NAFLD burden.

## Introduction

Nonalcoholic fatty liver disease (NAFLD), characterized by the accumulation of fat (hepatic steatosis) in more than 5% of hepatocytes, is currently recognized as an important driver leading to an increasing burden of chronic liver disease (CLD) worldwide and thus far lacks effective pharmacological therapies [1-4]. Among individuals with NAFLD, some will develop nonalcoholic steatohepatitis (NASH) and potentially progress to end-stage liver cirrhosis and carcinoma, with possible requirements for liver transplants and a poor prognosis [5-8]. In this context, many articles have investigated the epidemic pattern and attributable risk factors for NAFLD [2,3,9] to provide beneficial references for the prevention and control of this disease and thereby alleviate the global and regional socioeconomic burden of NAFLD.

In fact, tremendous heterogeneity of the NAFLD burden is observed around the world, and NAFLD has become the most rapidly growing contributor to liver mortality and morbidity [10]. Substantial NAFLD burdens have been reported successively in Asia, the Middle East, North Africa, Canada, and the United Kingdom [2,11-13], all of which indicate an urgent need for the systematic management and control of this disease. In addition to the liver insult itself, concomitant complications and comorbidities in other organs and systems related to NAFLD have likewise been investigated intensely [14]. The development of NAFLD necessitates the retention of intrahepatic triacylglycerol (IHTAG), whereas IHTAG has been reported to be strongly associated with obesity, insulin resistance, and diabetes mellitus type 2 (DM2) [15]. Self-evident associations between NAFLD and these disorders have been demonstrated in the clinic, and NAFLD patients tend to have DM2, dyslipidemia, and hypertension, which increase the susceptibility to cardiovascular complications [14,16,17]. The common factors contributing to the death of patients with NAFLD are often manifested in various complications, such as stroke and cardiovascular emergencies [18]. Accordingly, a greater understanding of the risk factors or possible comorbidities of NAFLD may help to decrease morbidity and mortality and thereby alleviate the disease burden.

To systematically and comprehensively grasp the global and regional socioeconomic burden of NAFLD during the past 30 years and to explore the interconnected diseases, we summarized the incidence, prevalence, mortality, and disability-adjusted life years (DALY) from the Global Burden of Disease 2019 (GBD 2019) study in this study. This updated epidemiologic pattern and potential risk factors for NAFLD are expected to benefit the development of efficient prevention and control policies, especially for those dedicated to the clinical treatment of NAFLD and public health care.

## Methods

### Data Acquisition

GBD 2019 provides the information about the burdens of 369 diseases and injuries along with 87 risk factors in the globe, different geographic areas, and 204 countries and territories [19]. Data on the NAFLD burden from 1990 to 2019, including its incidence, prevalence, death, DALY, and their corresponding age-standardized rates (ASRs), were acquired from the Global Health Data Exchange GBD results tool [20]. Meanwhile, information about the distributions of sex and age and related comorbidities, including DM2, stroke, and ischemic heart disease (IHD), was also obtained. The rates were standardized according to the GBD world population and were reported per 100,000 person-years. For the current report, we used the GBD results tool to extract the estimates and their 95% certainty intervals (CIs) for the prevalence of cases, deaths, and DALYs as measures of the NAFLD burden from 1990 to 2019 by region and country. To better exhibit the age distribution of the NAFLD burden, the patients were classified into 3 groups, namely, those aged 15 to 49 years, 50 to 69 years, and above 70 years (no data for those under 15 years). The sociodemographic index (SDI) is a composite indicator of social background and economic conditions that influence health outcomes in each location. In short, it is the geometric mean of 0 to 1 indices of total fertility rate for those younger than 25 years old, mean education for those 15 years old and older, and lag‐distributed income per capita [19], which has been reported to be correlated with the incidence and mortality of diseases. Based on that, 204 countries and their territories were classified into 5 groups according to the SDI values calculated in each year from 1990 to 2019 (low-SDI, low-middle-SDI, middle-SDI, high-middle-SDI, and high-SDI; Multimedia Appendix 1), to explore the association between NAFLD burden and social development degrees in different regions.

### Statistical Analysis

The incidence, prevalence, death, and DALYs and their corresponding ASRs were the main metrics characterizing the NAFLD burden and were compared at the global, regional, and country levels. CIs were calculated from 1000 estimates for each parameter, and 95% CIs were defined by the 25th and 975th values of the ordered 1000 estimates; 95% CI excluding 0 was considered statistically significant. To investigate the dynamic changes in the NAFLD burden, we further calculated the estimated annual percentage change (EAPC) to delineate the temporal trend in different ASRs for the NAFLD burden. Moreover, we constructed a regression model fitting the natural logarithm of the ASR with the calendar year, namely, ln (ASRs) = α+ β× calendar year + ε, to estimate the EAPC with its 95% CI based on the formula of 100 × (exp [β] − 1). If the EAPC value and its 95% CI were both above zero, the changed trend of ASR was considered upwards and vice versa. Otherwise, the ASR was considered stable over time [21]. Finally, we examined the association between the ASRs of NAFLD and the corresponding SDI value of each year using Pearson correlation analysis, as well as associations between the incidence or prevalence and DM2/stroke/IHD. All statistical analyses were performed using GraphPad Prism (version 8; GraphPad Software).

### Ethics Approval

Ethics approval was waived, as all the data in this study was obtained from GBD 2019 study.

## Results

### Incidence of NAFLD

**Figure 1:**
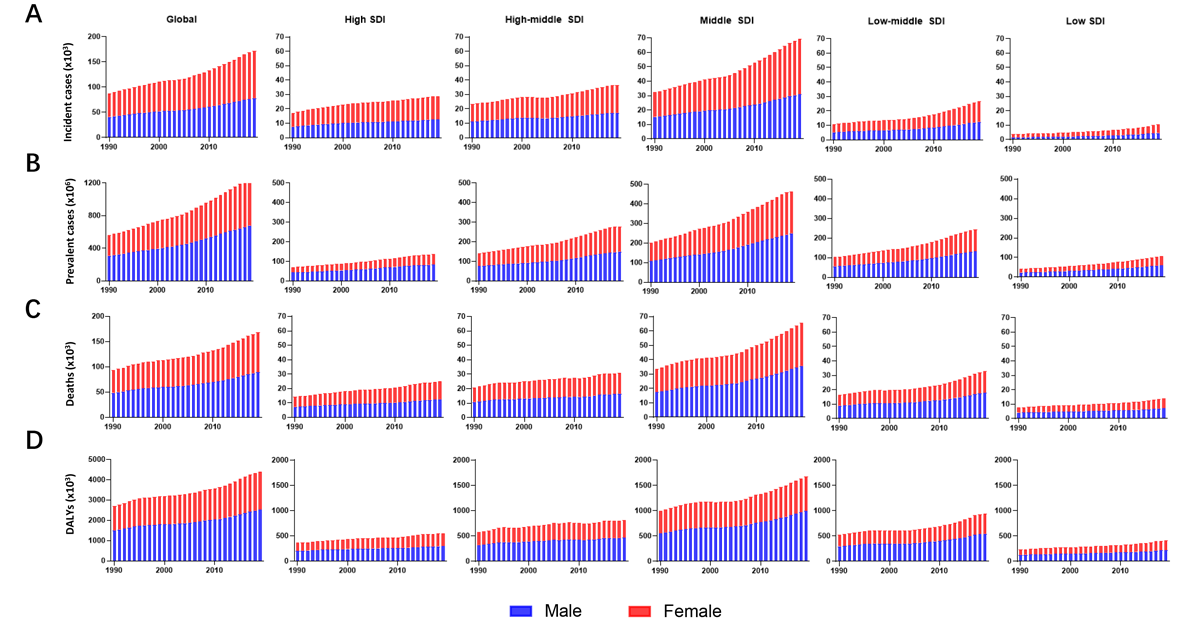
The trends of changes in NAFLD incidence, prevalence, deaths, and DALY from 1990 to 2019. The trends of changes in (A) incidences, (B) prevalence, (C) deaths, and (D) DALY (D) are shown. Blue bars represent males, and red bars represent females. DALYs: disability-adjusted life years; NAFLD: nonalcoholic fatty liver disease; SDI: sociodemographic index.

Globally, the incidence of NAFLD increased sharply in the past 30 years from 88,180 (95% CI 62,300-128,320) in 1990 to 172,330 (95% CI 125,780-243,640) in 2019, while there were no obvious changes after standardization by age (EAPC 0.1, 95% CI 0.04 to 0.23; Table S1 in Multimedia Appendix 2). As shown in Figure 1A and Table S1 in Multimedia Appendix 2, there were elevations in the incidence in both sexes, with a slightly higher incidence in women. Meanwhile, various SDI regions presented with gradually increasing NAFLD incidences, which mainly affected the low-middle (from 10,950 in 1990 to 26,640 in 2019) and middle SDI regions (from 32,460 in 1990 to 69,670 in 2019). However, there were no clear changes in age-standardized incidence rate (ASIR) among different SDI regions or by sex (Image A in Multimedia Appendix 3).

**Figure 2:**
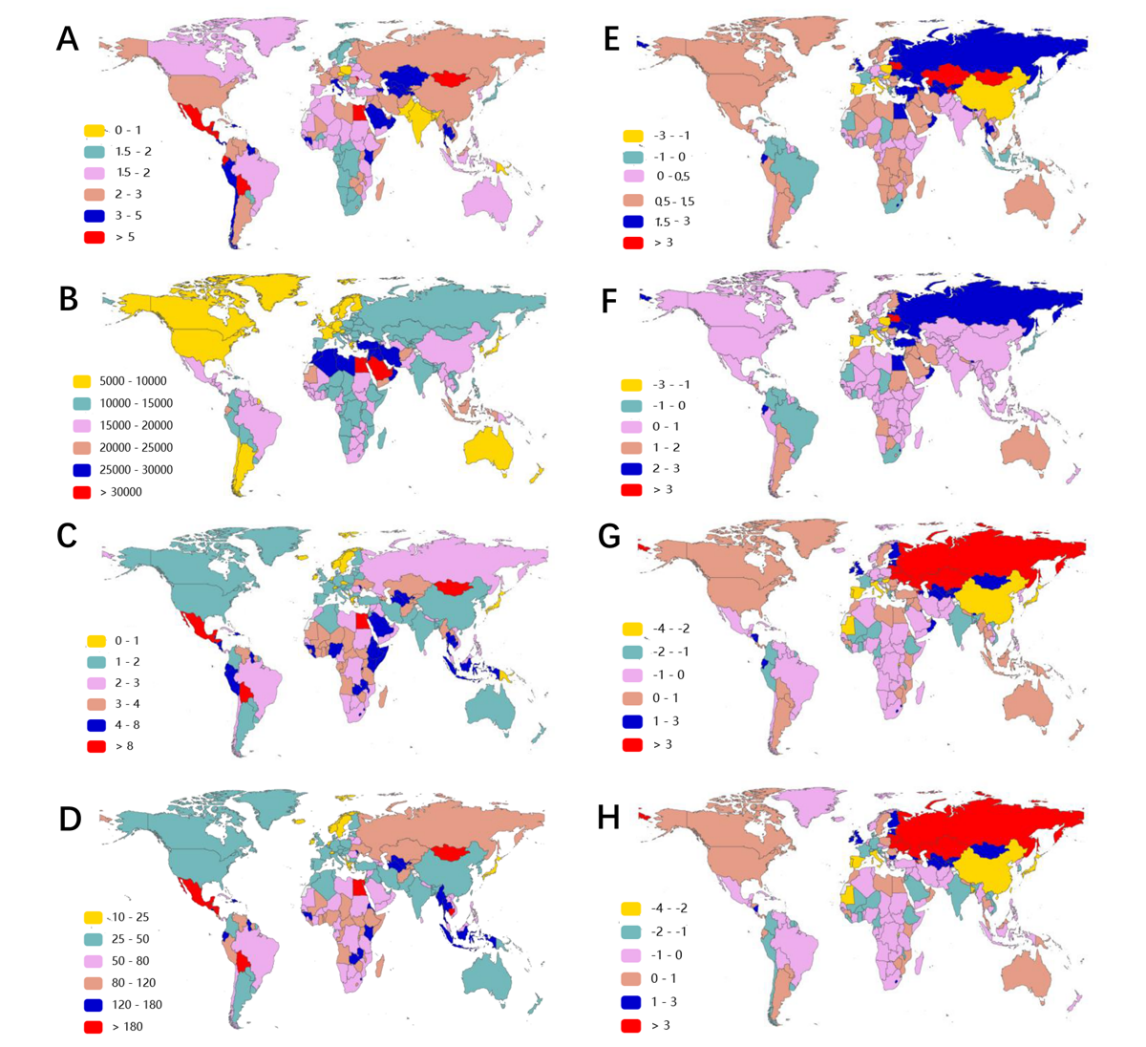
The age-standardized rates of NAFLD in 2019 and EAPC of NAFLD ASRs from 1990 to 2019 in 204 countries and territories. The (A) ASIR, (B) ASPR, (C) ASDR, and (D) age-standardized DALY of NAFLD around the world in 2019 are shown. The EAPC of (E) ASIR, (F) ASPR, (G) ASDR, and (H) age-standardized DALY in the past 30 years are shown. ASDR: age-standardized death rate; ASIR: age-standardized incident rate; ASPR: age-standardized prevalent rate; ASRs: age-standardized rates; DALY: disability-adjusted life years; EAPC: estimated annual percentage change; NAFLD: nonalcoholic fatty liver disease.

**Figure 3:**
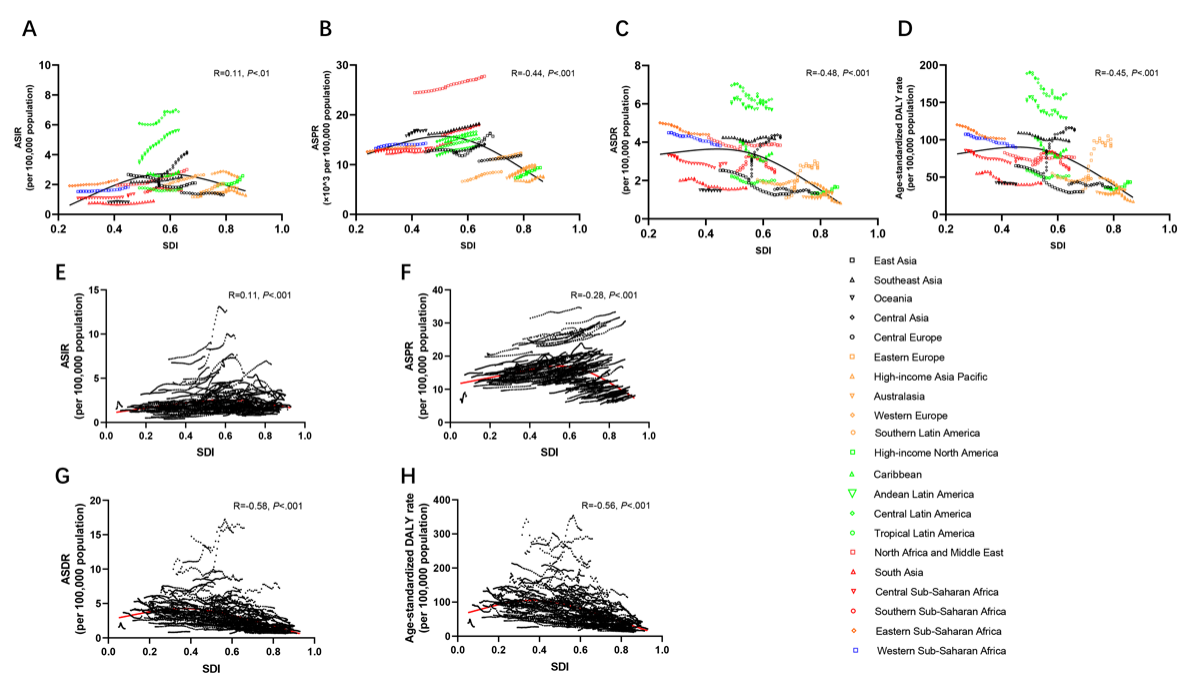
Correlation analyses between ASRs of NAFLD and SDI in 21 regions and 204 territories from 1990 to 2019. The SDI presented no obvious correlation with the (A, E) ASIR and negative correlations with (B, F) ASPR, (C, G) ASDR, and (D, H) age-standardized DALY in 21 regions and 204 territories. ASDR: age-standardized death rate; ASIR: age-standardized incident rate; ASPR: age-standardized prevalent rate; ASR: age-standardized rate; DALY: disability-adjusted life years; NAFLD: nonalcoholic fatty liver disease; SDI: sociodemographic index.

Although the overall incidences displayed upward conditions, ASIR of NAFLD and its changed trend presented immense heterogeneity among different countries and territories (Figure 2A,E; Table S1 in Multimedia Appendix 2). Specifically, the top 3 ASIRs of NAFLD were Central Latin America (6.88 per 100,000 population), Andean Latin America (5.62 per 100,000 population), and Central Asia (4.19 per 100,000 population). Central Asia, Eastern Europe, and the Middle East presented with great increases in ASIR changes during the past 30 years, with relatively higher EAPCs of ASIRs. East Asia had a negative change trend in ASIR (Figure 2E). Furthermore, from the country’s perspective, Mongolia had the highest ASIR in 2019 (12.65 per 100,000 population), and Papua New Guinea had the lowest (0.45 per 100,000 population). Finally, we analyzed the associations between SDI and ASIR among 21 regions (*r*=0.11, *P*<.01) and 204 countries (*r*=0.11, *P*<.001), which presented no obvious correlations (Figure 3A,E).

### NAFLD Prevalence

In the past 3 decades, the prevalence of NAFLD increased by more than 2-fold at the global level from 561,370,000 (95% CI 498,430,000-633,300,000) in 1990 to 1,235,700,000 (95% CI 1,109,540,000-1,378,530,000) in 2019 (Table S2 in Multimedia Appendix 2). EAPC, indicating the temporal trend of NAFLD age-standardized prevalence rate (ASPR), also presented significant upregulation (0.77, 95% CI 0.69-0.85). There were no marked differences between sexes or among various SDI regions regarding the prevalence and its change trend, all showing obvious upward changes (Figure 1B; Image B in Multimedia Appendix 3, and Table S2 in Multimedia Appendix 2).

In Table S2 in Multimedia Appendix 2, east Asia, south Asia, north Africa, and the Middle East maintained the highest prevalence globally in 1990 and 2019. However, the top 3 prevalences after age standardization were in north Africa and the Middle East (27,750 per 100,000 population), southeast Asia (18,300 per 100,000 population), and southern Sub-Saharan Africa (18,080 per 100,000 population; Figure 2B; Table S2 in Multimedia Appendix 2). Meanwhile, the regions with a higher EAPC of ASPR were around the Mediterranean (Figure 2F). Egypt had the highest ASPR in 2019 among all countries (34.69 per 100,000 population). Moreover, there were slightly negative correlations between ASPR and SDI among 21 regions (*r*=0.44, *P*<.001) and 204 countries (*r*=0.28, *P*<.001; Figures 3B and 3F), which demonstrated that the more developed the region was, the lower the ASPR.

### NAFLD-Related Mortalities

Although the global deaths due to NAFLD increased by almost 2-fold (from 93,760 in 1990 to 168,970 in 2019), the age-standardized death rate appeared to descend from 2.39 per 100,000 population (95% CI 1.84-3.05) in 1990 to 2.09 per 100,000 population (95% CI 1.61-2.60) in 2019, as demonstrated by the EAPC of ASDR with a negative value (0.67, 95% CI 0.76 to 0.57; Table S3 in Multimedia Appendix 2). After the classification by sex and SDI value, the cases of death sharply increased and the ASDR visibly decreased among all groups, which is similar to that observed at the global level (Figure 1C, Image C in Multimedia Appendix 3, and Table S3 in Multimedia Appendix 2).

Throughout various regions, NAFLD mortality and ASDR revealed extreme variations. East and south Asia had the highest mortality in both 1990 and 2019, while the top 3 ASDRs were Central Latin America, Andean Latin America, and eastern Sub-Saharan Africa (Figure 2C; Table S3 in Multimedia Appendix 2). However, owing to the relatively high ASDR in 1990, the temporal changes displayed negative trends in most regions. The most significant reductions in ASDR were observed in east Asia (EAPC 3.04, 95% CI 3.4 to 2.69), high-income Asia Pacific (EAPC 1.66, 95% CI 1.97 to 1.34), and western Europe (EAPC 1.32, 95% CI 1.41 to 1.24; Figure 2G and Table S3 in Multimedia Appendix 2). Similar to ASPR, Egypt had the highest ASDR in 2019 (15.97 per 100,000 population). In addition, correlation analyses demonstrated that SDI had a negative association with ASDR among 21 regions (*r*=0.48, *P*<.001) and 204 countries (*r*=0.58, *P*<.001) (Figures 3C and 3G), which might indicate higher ASDR in developing territories.

### NAFLD DALY

DALY is a critical parameter assessing disease burden, including years of life lost (YLL) owing to premature death and years lived with disability (YLD). As shown in Table S4 in Multimedia Appendix 2, global DALYs regarding NAFLD were elevated from 2,711,270 (95% CI 2,078,580-3,478,940) to 4,417,280 (95% CI 3,348,220-5,671,200). However, age-standardized DALYs presented decreased changes, with an EAPC of 0.82 (95% CI 0.93 to 0.70). In Figure 1D, the DALYs in both sexes and among different SDI regions exhibited obvious increases. However, the age-standardized DALYs all presented decreasing trends, with a more significant decline in women relative to men and in the middle SDI region relative to other SDI regions (Image D in Multimedia Appendix 3 and Table S4 in Multimedia Appendix 2).

Similar to the epidemiologic pattern of NAFLD-related death, the highest DALYs affected east and south Asia. The top 3 age-standardized DALYs are in Central Latin America (161.39 per 100,000 population), Andean Latin America (128.67 per 100,000 population), and Central Asia (112.39 per 100,000 population) (Figure 2D). The decline in age-standardized DALY was most pronounced in east Asia (EAPC 3.42, 95% CI 3.82 to 3.03), the high-income Asia Pacific (EAPC 2.15, 95% CI 2.48 to 1.81), and western Europe (EAPC 1.62, 95% CI 1.71 to 1.53), whereas eastern Europe and central Asia presented significant elevations (Figure 2H and Table S4 in Multimedia Appendix 2). Finally, we found that age-standardized DALY had a marked negative correlation with SDI among 21 territories (*r*=0.45, *P*<.001) (Figure 3D) or among 204 countries (*r*=0.56, *P*<.001) (Figure 3H).

### Age Distribution

**Figure 4:**
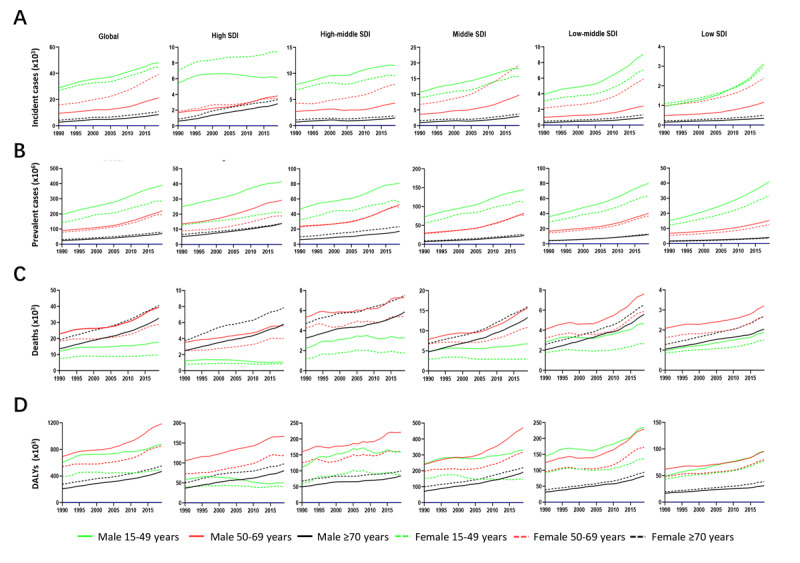
The trends of changes in NAFLD incidence, prevalence, deaths, and DALYs from 1990 to 2019 in different age groups. The trends of changes in (A) incidence, (B) prevalence, (C) deaths, and (D) DALYs. DALY: disability-adjusted life years; NAFLD: nonalcoholic fatty liver disease; SDI: sociodemographic index.

As depicted in Figure 4, all age groups presented gradually increasing trends in incidence, prevalence, death, and DALY, especially in low-middle SDI regions, regardless of sex and SDI values. Nevertheless, there was a certain heterogeneity in the specific age distribution. Young adults (aged from 15 to 49 years) dominated the NAFLD incidence and prevalence over the past 30 years, with global male incidence changing from 28,785 in 1990 to 47,862 in 2019 and with global male prevalence changing from 195,268,990 in 1990 to 388,787,021 in 2019 (Figures 4A and 4B and Multimedia Appendix 4), while the incidence and prevalence among females were slightly less than those among males. Interestingly, in high-SDI regions, NAFLD incidence was always higher in females than in males (females: 6,847 in 1990 to 9555 in 2019; males: 5399 in 1990 to 6082 in 2019; Figure 4A and Multimedia Appendix 4). In terms of NAFLD-related deaths, those aged 50 to 69 years were mostly men, and the elderly (aged above 70 years) held the dominant place among women, whereas young adults had relatively low mortality, which might correlate with the chronic and slow progression of NAFLD (Figure 4C). The NAFLD DALYs mainly influenced the quinquagenarian males with highest DALYs in 2019 of 1,186,057, but in relatively underdeveloped regions, the DALYs of young adults remained close to the quinquagenarians (Figure 4D and Multimedia Appendix 4). Consequently, age may be a vital factor affecting the NAFLD burden in various regions.

### Associations Between NAFLD and Other Interconnected Diseases

**Figure 5:**
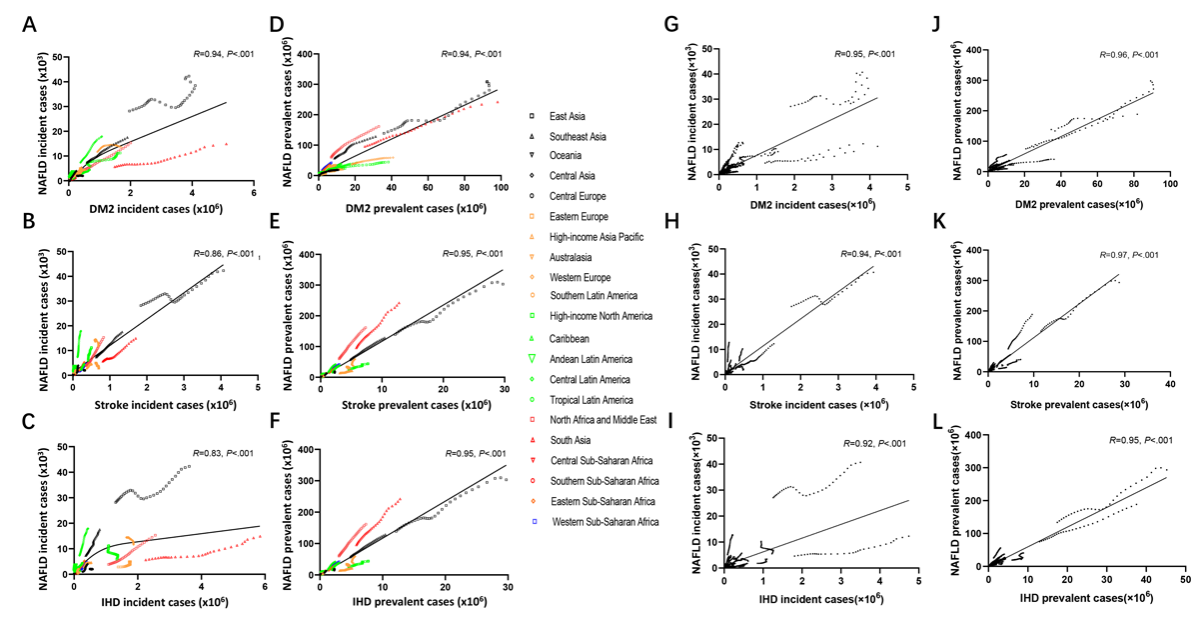
The associations of incidence and prevalence between NAFLD and other comorbidities in 21 regions and 204 territories from 1990 to 2019. NAFLD presented strongly positive correlations of incidence/prevalence with (A, D, G, J) DM2, (B, E, H, K) stroke, and (C, F, I, L) IHD in 21 regions and 204 territories from 1990 to 2019. DM2: diabetes mellitus type 2; IHD: ischemic heart disease; NAFLD: nonalcoholic fatty liver disease.

To further explore this hazardous disease, we analyzed the associations between NAFLD and 3 common interconnected disorders [22]. Interestingly, we found that the incidence of NAFLD presented strongly positive correlations with that of DM2, stroke, and IHD, both in 21 territories (*r*=0.94, *P*<.001; *r*=0.86, *P*<.001; *r*=0.83, *P*<.001; Figures 5A-5C) and 204 countries (*r*=0.95, *P*<.001; *r*=0.94, *P*<.001; *r*=0.92, *P*<.001; Figures 5G-5I). Meanwhile, the same positive associations were observed in the prevalence of NAFLD and DM2, stroke, and IHD in 21 territories (*r*=0.94, *P*<.001; *r*=0.95, *P*<.001; *r*=0.95, *P*<.001; Figures 5D-5F) and in 204 countries (*r*=0.96, *P*<.001; *r*=0.97, *P*<.001; *r*=0.95, *P*<.001; Figures 5J-5L).

## Discussion

### Principal Findings

In this study, we comprehensively assessed the global burden of NAFLD and compared its associations with common correlative diseases. In general, the increasing disease burden caused by NAFLD has placed heavy pressure on contemporary society at a gradual pace over recent decades, with corresponding epidemiological parameters showing upward changes, including increases in incidence, prevalence, deaths, and DALYs. A previous study [23] suggested a significant shift in NAFLD burden toward a younger population, which was echoed by our findings. However, after age standardization, there were no obvious upregulations in NAFLD incidence, even though declining alterations in ASDR and age-standardized DALYs were observed. Meanwhile, we uncovered distinctly negative correlations between the SDI and ASPR, ASDR, and age-standardized DALYs. Finally, we confirmed 3 strongly relevant diseases accompanied by NAFLD incidence and prevalence, namely, DM2, stroke, and IHD. Therefore, the systematic understanding of global epidemiologic patterns for NAFLD and its interrelated disorders may be valuable for the development of corresponding prevention and control strategies, especially for public health policy makers and clinical physicians.

Compared to 1990, the overall incidence and prevalence of NAFLD in 2019 increased by approximately 2-fold, mainly impacting low- and middle-SDI regions, such as some countries in the Middle East and north Africa [2,24]. Furthermore, the same rising socioeconomic burden of NAFLD has influenced relatively developed regions. Williams reported that the prevalence of NAFLD was up to 46% in the United States [25], while other European countries, including Italy, Greece, and the United Kingdom, presented with a markedly increased incidence and prevalence, resulting in an increasing socioeconomic burden [26-28]. In addition, relatively young patients dominated NAFLD morbidity and their prevalence rapidly increased, especially in low-middle-SDI regions. Given the pathogenesis of NAFLD associated with fat accumulation in hepatocytes and the growing obesity among youngsters [1,29], this seems reasonable to explain the increasing number of young patients. Interestingly, unlike the increased number of NAFLD cases, ASIR presented no marked upregulation, which might partly be associated with changes in sociodemographic structure in different areas and countries [30,31]. The reason behind it remains under investigation and requires more research.

NAFLD is a complex and multifactor disorder that is affected by metabolic and environmental factors, along with genetic and epigenetic predispositions involving multiple organs and diverse mechanisms [32]. The exact contribution of each factor to the development of NAFLD is unknown, requiring further investigation, and it may vary by geographic location, which is associated with the great heterogeneity of the NAFLD prevalence in different districts. Recently, metabolic imbalances have been gradually considered the predominant risk factor, and an international expert group has agreed to change the name of NAFLD to metabolic (dysfunction)–associated fatty liver disease (MAFLD) [33,34]. DM2, as the most prevalent metabolic disease worldwide, was found, in our study, to correlate significantly with the NAFLD incidence and prevalence, which is concurrent with previous studies [35,36] and further highlights the vital role of metabolic dysfunction in NAFLD. Meanwhile, owing to alterations in the diet structure in modern life, populations of individuals with obesity are increasing at a rapid pace, which is regarded as the main risk factor for diabetes and fuels NAFLD-related morbidity. In fact, NAFLD may in turn be a pathogenic component of the development of DM2, and the bidirectional relationship between NAFLD and type 2 diabetes remains controversial and needs more exploration [37]. However, active prevention and control of obesity and diabetes can help alleviate the development of NAFLD to some extent [38,39].

NAFLD poses a substantial threat to individual health, with the number of deaths and DALYs increasing dramatically from 1990 to 2019, which was primarily driven by population growth and aging worldwide, specifically in low-middle–income countries [30]. In the meantime, the patients older than 50 years had the most deaths and DALYs, regardless of sex or the different SDI regions, which could be expected because of the aging population and the worse response to therapy among the elderly population. However, the age-standardized death and DALY rates were decreased globally and were particularly lower in higher-SDI regions. With rapid progression of society and elevation of health care, we primarily regard that the early diagnosis and prompt treatment could improve the survival and prognosis of patients with NAFLD. Additionally, Allen et al [23] previously reported the shift in NAFLD incidence toward a younger population who accounted for the majority of patients with NAFLD , which was consistent with our results. Meanwhile, considering the chronic course of NAFLD [40], it could be easily concluded that there showed relatively low deaths and DALY in young adults. Therefore, the global ASDR and age-standardized DALY presented downward changes after age standardization. Besides, the association analyses showed significantly negative relations between SDI and ASDR or age-standardized DALY, indicating a more serious NAFLD burden in lower-income regions. In specific, we observed relatively high ASDR and age-standardized DALY in Latin America, north Africa, and the Middle East. High-income Asia Pacific, Central Europe, and high-income North America had lower ASDRs and age-standardized DALY. Differences in access to health care and medical level have remained the fundamental factor for immense heterogeneity in the disease’s mortality across countries [41]. Therefore, most NAFLD-related deaths could partly be reduced in high-income countries through easy access to better health care and a stronger health infrastructure, such as early-stage identification of NAFLD and education of patients. Of course, other factors contributing to the decreases in the ASRs of NAFLD death and DALYs remained to be investigated.

In the general population, more than 10% of all patients with NAFLD may develop NASH [42], which is characterized by steatosis, hepatocellular ballooning, lobular inflammation, and often fibrosis [43]. During the response to tissue damage, hepatocytes are replaced by type I collagen produced by stellate cells, leading to the progression of NASH toward fibrosis and cirrhosis with overt clinical consequences [44-46]. Furthermore, patients with NASH have been reported to be highly susceptible to liver cancer [47]. Failure to recognize high-risk individuals and provide prompt treatment for NAFLD might lead to progression to NASH and even cirrhosis or carcinoma with a poor prognosis and high mortality, especially in low-income countries. Meanwhile, it also partly explained why the ASDR and age-standardized DALY of NAFLD were lower in regions with higher SDI values than in those with lower SDI values. Accordingly, to decrease the mortality and DALYs of NAFLD, preventing its development into NASH and then cirrhosis or liver cancer is essential.

In addition to hepatic causes, cardiovascular disease is the leading cause of death in patients with NAFLD, with mortality up to approximately 20% [48]. In this study, we showed that common cardiovascular diseases, including stroke and ischemic heart disease, had strong positive associations with the incidence and prevalence of NAFLD, which can be attributable to shared risk factors between these 2 diseases, such as dyslipidemia, insulin resistance, hypertension, and obesity [49,50]. Consequently, co-occurring stroke and IHD in patients with NAFLD merits considerable attention for better prevention of cardiovascular events and lower mortality.

### Limitations

There remain some obvious limitations of this study. First, we relied heavily on GBD estimates for this study. The accuracy of the GBD estimates was limited by the quality and availability of each country’s vital registration system and a mass of undefined NAFLD cases in their registry data. Moreover, other possible intermediate factors or confounders during the correlation analyses were not included for adjustment owing to lack of data on other relevant parameters. Other potential interconnected diseases with NAFLD, such as dyslipidemia that are unavailable in GBD 2019 database, await further investigation. In addition, we preliminarily conducted the association analyses and calculated the potential correlation coefficients via the GBD data set but cannot perform the validation on correlation coefficients due to lacking other analogous study populations covering almost all regions and countries in the world. Finally, subgroup analyses were not performed among patients with NAFLD grouped on the basis of whether they had comorbid metabolic syndrome, were on medication therapy, or had other factors.

### Conclusions

The global burden of NAFLD is gradually increased and is predicted to continue to increase in the future. The morbidity presented a clear shift toward young populations. Meanwhile, higher age-standardized death and DALY rates can be observed in aged individuals and low-SDI regions. Furthermore, NAFLD presented strong correlations with three high-risk comorbidities, namely, DM2, stroke, and ischemic heart disease. Therefore, the development of cost-effective global and regional strategies to mitigate NAFLD morbidity and mortality, alleviate the socioeconomic burden, and prevent risky interconnected diseases are urgently required by policy makers and clinical physicians.

## Appendix

Multimedia Appendix 1 Original data for Fig. 3.

Multimedia Appendix 2 Supplementary Tables.

Multimedia Appendix 3 The change trends of NAFLD ASIR, ASPR, ASDR and age-standardized DALYs from 1990 to 2019. The change trends of ASIR (A), ASPR (B), ASDR (C) and age-standardized DALYs (D) were displayed. Blue bars represent males and red bars represent females. NAFLD: nonalcoholic fatty liver disease; ASIR: age-standardized incident rate; ASPR: age-standardized prevalent rate; ASDR: age-standardized death rate; DALYs: disability-adjusted life years; SDI: social-demographic index.

Multimedia Appendix 4 Original data for Fig. 4.

## Abbreviations

ASIR: age-standardized incidence rate
ASR: age-standardized rate
ASPR: age-standardized prevalence rate
CI: certainty interval
CLD: chronic liver disease
DALY: disability-adjusted life years
DM2: diabetes mellitus type 2
EAPC: estimated annual percentage change
GBD 2019: Global Burden of Disease 2019
IHD: ischemic heart disease
IHTAG: intrahepatic triacylglycerol
MAFLD: metabolic (dysfunction)–associated fatty liver disease
NAFLD: nonalcoholic fatty liver disease
NASH: nonalcoholic steatohepatitis
SDI: sociodemographic index
YLD: years lived with disability
YLL: years of life lost

## Data Availability

The data sets used in the present study are available in the GBD 2019 study.
