# Supplementary material for: The Global, Regional, and National Burden and Trends of NAFLD in 204 Countries and Territories: An Analysis From Global Burden of Disease 2019
Source: JMIR Public Health Surveill. 2022 Dec 12;8(12):e34809. doi: 10.2196/34809 (PMC9793331; doi:10.2196/34809)
Supplement: Multimedia Appendix 2 [file publichealth_v8i12e34809_app2.docx]

| **Table S1.** The incidence of NAFLD in 1990/2019 and temporal trends | | | | | | | | | | | | | | | | |
| --- | --- | --- | --- | --- | --- | --- | --- | --- | --- | --- | --- | --- | --- | --- | --- | --- |
| Characteristics | 1990 | | | | | | |  | 2019 | | | | | | | 1990-2019 |
|  | Incident cases No×10^3^ (95% CI) | |  | | ASIR/10^5^ No. (95% CI) | | |  | Incident cases No×10^3^ (95% CI) | |  | | ASIR/10^5^ No. (95% CI) | | | EAPC No. (95% CI) |
| Overall | 88.18(62.3-128.32) | |  | | 1.94(1.38-2.77) | | |  | 172.33(125.78-243.64) | |  | | 2.08(1.52-2.93) | | | 0.1(-0.04-0.23) |
| Sex |  | |  | |  | | |  |  | |  | |  | | |  |
| Male | 40.98(28.48-61.17) | |  | | 1.79(1.27-2.56) | | |  | 77.87(55.78-111.24) | |  | | 1.93(1.4-2.74) | | | 0.06(-0.07-0.2) |
| Female | 47.19(33.5-66.22) | |  | | 2.08(1.48-2.93) | | |  | 94.46(67.94-132.2) | |  | | 2.23(1.6-3.12) | | | 0.12(-0.02-0.26) |
| Socio-demographic factor |  | |  | |  | | |  |  | |  | |  | | |  |
| High SDI | 17.35(12.13-25.24) | |  | | 1.87(1.3-2.75) | | |  | 28.92(21.91-39.2) | |  | | 2.16(1.58-3.06) | | | 0.31(0.22-0.41) |
| High-middle SDI | 23.49(16.69-34.07) | |  | | 2.08(1.48-2.94) | | |  | 36.64(26.06-53.1) | |  | | 1.99(1.41-2.92) | | | -0.28(-0.44--0.12) |
| Middle SDI | 32.46(22.8-46.32) | |  | | 2.49(1.8-3.44) | | |  | 69.67(50.16-98.09) | |  | | 2.58(1.9-3.57) | | | 0.04(-0.14-0.21) |
| Low-middle SDI | 10.95(7.51-15.99) | |  | | 1.4(0.99-2) | | |  | 26.64(18.85-38.65) | |  | | 1.66(1.19-2.39) | | | 0.40(0.15-0.64) |
| Low SDI | 3.87(2.53-5.74) | |  | | 1.28(0.85-1.89) | | |  | 10.51(7.25-15.58) | |  | | 1.5(1.04-2.19) | | | 0.41(0.28-0.54) |
| Region |  | |  | |  | | |  |  | |  | |  | | |  |
| East Asia | 28.17(20.36-39.05) | |  | | 2.67(1.96-3.65) | | |  | 42.27(29.93-59.58) | |  | | 2.1(1.51-2.93) | | | -1.26(-1.64--0.88) |
| Southeast Asia | 7.32(4.89-10.8) | |  | | 2.15(1.44-3.14) | | |  | 17.48(12.61-24.54) | |  | | 2.54(1.86-3.52) | | | 0.55(0.49-0.62) |
| Oceania | 0.04(0.02-0.05) | |  | | 0.81(0.58-1.13) | | |  | 0.08(0.06-0.12) | |  | | 0.82(0.6-1.13) | | | -0.01(-0.09-0.06) |
| Central Asia | 0.99(0.65-1.49) | |  | | 1.81(1.18-2.74) | | |  | 3.96(2.66-5.83) | |  | | 4.19(2.87-6.08) | | | 3.17(3.06-3.28) |
| Central Europe | 1.97(1.43-2.79) | |  | | 1.45(1.04-2.06) | | |  | 1.97(1.39-2.82) | |  | | 1.3(0.89-1.93) | | | -0.2(-0.31--0.1) |
| Eastern Europe | 2.86(1.81-4.58) | |  | | 1.18(0.74-1.88) | | |  | 4.93(3.04-7.94) | |  | | 2.04(1.22-3.32) | | | 2.34(2.19-2.49) |
| High-income Asia Pacific | 2.84(2.18-3.78) | |  | | 1.39(1.07-1.84) | | |  | 4.59(3.64-5.81) | |  | | 1.27(0.99-1.65) | | | -0.59(-0.88--0.29) |
| Australasia | 0.31(0.21-0.44) | |  | | 1.4(0.95-2) | | |  | 0.65(0.48-0.87) | |  | | 1.74(1.27-2.4) | | | 1.02(0.86-1.18) |
| Western Europe | 11.66(7.93-17.37) | |  | | 2.71(1.82-4.05) | | |  | 13.55(9.72-19.42) | |  | | 2.46(1.7-3.62) | | | -0.43(-0.56--0.3) |
| Southern Latin America | 0.91(0.58-1.39) | |  | | 1.96(1.24-2.99) | | |  | 1.98(1.29-3.01) | |  | | 2.65(1.72-4.06) | | | 0.98(0.91-1.05) |
| High-income North America | 5.97(3.94-9.34) | |  | | 1.9(1.24-2.95) | | |  | 11.22(8.1-15.71) | |  | | 2.58(1.81-3.79) | | | 0.81(0.61-1.02) |
| Caribbean | 0.79(0.56-1.13) | |  | | 2.74(1.95-3.87) | | |  | 1.47(1.01-2.13) | |  | | 2.9(2-4.22) | | | 0.08(-0.06-0.22) |
| Andean Latin America | 0.89(0.62-1.31) | |  | | 3.44(2.39-4.97) | | |  | 3.42(2.34-4.87) | |  | | 5.62(3.86-7.97) | | | 1.7(1.56-1.83) |
| Central Latin America | 7.34(4.79-11.11) | |  | | 6.11(4.03-9.06) | | |  | 17.86(12.13-25.98) | |  | | 6.88(4.7-9.98) | | | 0.64(0.53-0.76) |
| Tropical Latin America | 2.24(1.38-3.5) | |  | | 1.77(1.08-2.72) | | |  | 4.4(2.79-6.58) | |  | | 1.72(1.1-2.56) | | | -0.24(-0.41--0.07) |
| North Africa and Middle East | 3.86(2.74-5.47) | |  | | 1.95(1.37-2.76) | | |  | 15.29(10.77-21.69) | |  | | 3.01(2.14-4.22) | | | 1.65(1.54-1.76) |
| South Asia | 5.63(3.5-8.73) | |  | | 0.76(0.5-1.12) | | |  | 14.86(9.9-22.53) | |  | | 0.91(0.62-1.33) | | | 0.34(0.02-0.67) |
| Central Sub-Saharan Africa | 0.36(0.23-0.56) | |  | | 1.09(0.7-1.69) | | |  | 1.2(0.77-1.82) | |  | | 1.37(0.89-2.1) | | | 0.56(0.37-0.75) |
| Eastern Sub-Saharan Africa | 1.9(1.18-2.89) | |  | | 1.91(1.18-2.93) | | |  | 5.35(3.49-8.07) | |  | | 2.31(1.48-3.5) | | | 0.56(0.47-0.66) |
| Southern Sub-Saharan Africa | 0.51(0.35-0.74) | |  | | 1.48(1-2.16) | | |  | 1.06(0.78-1.47) | |  | | 1.61(1.2-2.17) | | | 0.01(-0.2-0.22) |
| Western Sub-Saharan Africa | 1.61(1.04-2.42) | |  | | 1.52(1-2.24) | | |  | 4.73(3.13-6.97) | |  | | 1.83(1.22-2.66) | | | 0.53(0.43-0.62) |
| Note: ASIR, age-standardized incident rate; SDI, social-demographic index; EAPC, estimated annual percentage change. | | | | | | | | | | | | | | | | |
| **Table S2.** The prevalence of NAFLD in 1990/2019 and temporal trends | | | | | | | | | | | | | | | |  |
| Characteristics | 1990 | | | | | | |  | 2019 | | | | | | | 1990-2019 |
|  | prevalence No×10^6^(95% CI) | |  | | ASPR×10^3^/10^5^ No. (95% CI) | | |  | prevalence No×10^6^ (95% CI) | |  | | ASPR×10^3^/10^5^ No. (95% CI) | | | EAPC No. (95% CI) |
| Overall | 561.37(498.43-633.3) | |  | | 12.07(10.78-13.54) | | |  | 1235.7(1109.54-1378.53) | |  | | 15.02(13.49-16.76) | | | 0.77(0.69-0.85) |
| Sex |  | |  | |  | | |  |  | |  | |  | | |  |
| Male | 309.5(274.83-348.28) | |  | | 13.42(12-15.04) | | |  | 679.29(610.68-753.74) | |  | | 16.79(15.14-18.61) | | | 0.76(0.66-0.86) |
| Female | 251.87(223.79-285.52) | |  | | 10.75(9.58-12.15) | | |  | 556.41(499.07-625.01) | |  | | 13.28(11.9-14.93) | | | 0.77(0.7-0.85) |
| Socio-demographic factor |  | |  | |  | | |  |  | |  | |  | | |  |
| High SDI | 72.14(64.38-81.12) | |  | | 7.64(6.8-8.61) | | |  | 138.64(125.51-153.33) | |  | | 10.53(9.43-11.73) | | | 1.21(1.17-1.26) |
| High-middle SDI | 141.26(126.2-158.55) | |  | | 12.27(10.96-13.75) | | |  | 278.09(251.21-308.48) | |  | | 15.34(13.8-17.1) | | | 0.78(0.66-0.89) |
| Middle SDI | 201.44(178.61-226.66) | |  | | 14.63(13.11-16.37) | | |  | 464.28(417.31-516.04) | |  | | 17.6(15.84-19.53) | | | 0.65(0.54-0.75) |
| Low-middle SDI | 103.56(91.62-117.47) | |  | | 12.94(11.52-14.69) | | |  | 245.75(219.06-276.67) | |  | | 15.23(13.66-17.08) | | | 0.55(0.46-0.64) |
| Low SDI | 42.62(37.51-48.45) | |  | | 12.87(11.42-14.52) | | |  | 108.18(95.42-122.65) | |  | | 14.28(12.74-16.05) | | | 0.34(0.31-0.37) |
| Region |  | |  | |  | | |  |  | |  | |  | | |  |
| East Asia | 138.89(122.56-157.83) | |  | | 12.54(11.11-14.23) | | |  | 303.13(272.38-339.5) | |  | | 15.68(14.02-17.55) | | | 0.81(0.52-1.1) |
| Southeast Asia | 56.61(50.13-63.62) | |  | | 16.11(14.46-18.02) | | |  | 128.07(114.73-142.46) | |  | | 18.3(16.51-20.31) | | | 0.45(0.44-0.47) |
| Oceania | 0.7(0.62-0.8) | |  | | 15.75(14.08-17.65) | | |  | 1.74(1.54-1.97) | |  | | 16.87(15.13-18.73) | | | 0.18(0.13-0.24) |
| Central Asia | 6.86(6.11-7.7) | |  | | 12.3(11.06-13.71) | | |  | 12.82(11.45-14.33) | |  | | 14.15(12.74-15.75) | | | 0.5(0.46-0.54) |
| Central Europe | 14.76(13.29-16.37) | |  | | 10.68(9.63-11.86) | | |  | 18.63(16.91-20.48) | |  | | 11.9(10.76-13.11) | | | 0.34(0.33-0.35) |
| Eastern Europe | 28.74(25.93-31.99) | |  | | 11.03(9.93-12.23) | | |  | 34.11(30.98-37.56) | |  | | 12.3(11.1-13.6) | | | 0.38(0.37-0.4) |
| High-income Asia Pacific | 13.54(12.04-15.25) | |  | | 6.84(6.08-7.69) | | |  | 20.94(18.78-23.28) | |  | | 7.67(6.83-8.63) | | | 0.56(0.45-0.67) |
| Australasia | 1.62(1.45-1.83) | |  | | 7.27(6.46-8.17) | | |  | 3.46(3.12-3.82) | |  | | 9.44(8.46-10.48) | | | 0.92(0.85-1) |
| Western Europe | 37.03(33.05-41.68) | |  | | 7.88(7.04-8.88) | | |  | 59.01(53.31-65.21) | |  | | 9.93(8.93-11.04) | | | 0.81(0.73-0.89) |
| Southern Latin America | 3.14(2.79-3.56) | |  | | 6.62(5.89-7.49) | | |  | 6.48(5.83-7.19) | |  | | 8.6(7.72-9.56) | | | 0.92(0.82-1.01) |
| High-income North America | 23.25(20.6-26.33) | |  | | 7.26(6.42-8.25) | | |  | 44.32(39.7-49.55) | |  | | 9.4(8.4-10.54) | | | 0.98(0.95-1.02) |
| Caribbean | 4.34(3.86-4.86) | |  | | 14.54(13.03-16.2) | | |  | 8.18(7.38-9.02) | |  | | 16.17(14.59-17.87) | | | 0.4(0.38-0.42) |
| Andean Latin America | 3.23(2.87-3.63) | |  | | 11.76(10.56-13.13) | | |  | 8.44(7.6-9.33) | |  | | 13.69(12.38-15.09) | | | 0.5(0.48-0.53) |
| Central Latin America | 17.06(15.12-19.2) | |  | | 14.59(13.11-16.25) | | |  | 42.17(37.82-46.84) | |  | | 16.62(14.96-18.39) | | | 0.43(0.41-0.44) |
| Tropical Latin America | 15.65(13.87-17.62) | |  | | 13.16(11.83-14.66) | | |  | 37.99(34.23-41.95) | |  | | 15.24(13.72-16.82) | | | 0.49(0.48-0.51) |
| North Africa and Middle East | 60.11(54.01-67.06) | |  | | 24.42(22.22-26.87) | | |  | 161.46(146.41-177.66) | |  | | 27.75(25.41-30.28) | | | 0.47(0.46-0.49) |
| South Asia | 95.44(83.69-108.91) | |  | | 12.25(10.87-13.94) | | |  | 241.84(214.85-273.69) | |  | | 14.51(12.97-16.4) | | | 0.54(0.42-0.65) |
| Central Sub-Saharan Africa | 4.25(3.71-4.87) | |  | | 12.57(11.11-14.25) | | |  | 11.34(9.89-13) | |  | | 13.33(11.8-15.1) | | | 0.16(0.15-0.18) |
| Eastern Sub-Saharan Africa | 13.97(12.17-15.98) | |  | | 12.56(11.14-14.17) | | |  | 36.09(31.61-41.13) | |  | | 13.71(12.22-15.37) | | | 0.29(0.28-0.3) |
| Southern Sub-Saharan Africa | 6.09(5.4-6.87) | |  | | 16(14.36-17.87) | | |  | 13.16(11.81-14.68) | |  | | 18.08(16.35-20) | | | 0.41(0.4-0.42) |
| Western Sub-Saharan Africa | 16.08(14.19-18.25) | |  | | 13.16(11.72-14.78) | | |  | 42.32(37.19-48.14) | |  | | 14.28(12.76-15.95) | | | 0.22(0.17-0.26) |
| Note: ASPR, age-standardized prevalent rate; SDI, social-demographic index; EAPC, estimated annual percentage change. | | | | | | | | | | | | | | | | |
| **Table S3.** The death of NAFLD in 1990/2019 and temporal trends | | | | | | | | | | | | | | |  | |
| Characteristics | | 1990 | | | | |  | | | 2019 | | | | | 1990-2019 | |
|  |  | Death  No×10^3^ (95% CI) | |  | | ASDR/10^5^ No. (95% CI) |  | | | Death No×10^3^ (95% CI) | |  | | ASDR/10^5^ No. (95% CI) | EAPC No. (95% CI) | |
| Overall | | 93.76(71.66-119.1) | |  | | 2.39(1.84-3.05) |  | | | 168.97(130.58-211.29) | |  | | 2.09(1.61-2.6) | -0.67(-0.76--0.57) | |
| Sex | |  | |  | |  |  | | |  | |  | |  |  | |
| Male | | 48.69(36.64-62.98) | |  | | 2.66(2.02-3.42) |  | | | 89.76(68.19-114.46) | |  | | 2.38(1.82-3.02) | -0.61(-0.71--0.52) | |
| Female | | 45.07(34.98-57.25) | |  | | 2.15(1.66-2.72) |  | | | 79.21(61.14-98.75) | |  | | 1.82(1.41-2.27) | -0.73(-0.82--0.64) | |
| Socio-demographic factor | |  | |  | |  |  | | |  | |  | |  |  | |
| High SDI | | 14.37(10.96-18.26) | |  | | 1.41(1.07-1.82) |  | | | 25.22(19.77-31.16) | |  | | 1.37(1.07-1.72) | -0.23(-0.31--0.14) | |
| High-middle SDI | | 21.02(16.16-26.38) | |  | | 2.02(1.56-2.53) |  | | | 31.22(24.09-39.33) | |  | | 1.57(1.22-1.97) | -1.08(-1.17--0.98) | |
| Middle SDI | | 33.93(26.37-42.47) | |  | | 3.51(2.73-4.42) |  | | | 65.53(50.28-82.54) | |  | | 2.8(2.17-3.52) | -0.92(-1.04--0.81) | |
| Low-middle SDI | | 16.5(12.28-21.39) | |  | | 2.79(2.09-3.63) |  | | | 32.89(24.84-42.44) | |  | | 2.47(1.88-3.16) | -0.67(-0.83--0.52) | |
| Low SDI | | 7.86(5.64-10.7) | |  | | 3.47(2.49-4.67) |  | | | 13.98(10.23-18.73) | |  | | 2.79(2.05-3.74) | -0.92(-0.99--0.85) | |
| Region | |  | |  | |  |  | | |  | |  | |  |  | |
| East Asia | | 21.51(17.07-26.73) | |  | | 2.53(2-3.09) |  | | | 25.41(19.63-31.41) | |  | | 1.29(1.01-1.58) | -3.04(-3.4--2.69) | |
| Southeast Asia | | 10.73(7.85-14.11) | |  | | 4.25(3.12-5.65) |  | | | 23.7(17.8-30.7) | |  | | 4.18(3.14-5.37) | 0.004 (-0.08-0.09) | |
| Oceania | | 0.05(0.03-0.07) | |  | | 1.51(1.11-2.05) |  | | | 0.11(0.07-0.15) | |  | | 1.46(1.04-1.96) | -0.07(-0.11--0.04) | |
| Central Asia | | 0.97(0.69-1.31) | |  | | 2.08(1.48-2.81) |  | | | 3.07(2.22-4.15) | |  | | 4.22(3.11-5.58) | 2.39(2.05-2.72) | |
| Central Europe | | 2.21(1.65-2.85) | |  | | 1.53(1.17-1.97) |  | | | 2.66(1.92-3.58) | |  | | 1.32(0.96-1.78) | -0.57(-0.71--0.42) | |
| Eastern Europe | | 2.98(2.16-4.01) | |  | | 1.09(0.8-1.44) |  | | | 8.17(5.89-10.98) | |  | | 2.65(1.91-3.56) | 3.36(2.75-3.98) | |
| High-income Asia Pacific | | 2.31(1.85-2.79) | |  | | 1.18(0.95-1.42) |  | | | 3.98(3.09-4.86) | |  | | 0.82(0.66-0.99) | -1.66(-1.97--1.34) | |
| Australasia | | 0.26(0.19-0.33) | |  | | 1.15(0.84-1.48) |  | | | 0.64(0.5-0.79) | |  | | 1.32(1.04-1.63) | 0.82(0.6-1.04) | |
| Western Europe | | 10.61(7.72-13.83) | |  | | 1.9(1.39-2.46) |  | | | 12.36(9.39-15.78) | |  | | 1.38(1.05-1.74) | -1.32(-1.41--1.24) | |
| Southern Latin America | | 0.94(0.65-1.29) | |  | | 2.05(1.45-2.78) |  | | | 1.63(1.17-2.18) | |  | | 1.96(1.42-2.64) | 0.09(-0.08-0.26) | |
| High-income North America | | 4.49(3.4-5.81) | |  | | 1.32(0.98-1.71) |  | | | 9.9(7.65-12.62) | |  | | 1.65(1.27-2.1) | 0.9(0.71-1.09) | |
| Caribbean | | 1.06(0.79-1.36) | |  | | 4.08(3.08-5.23) |  | | | 1.77(1.25-2.43) | |  | | 3.43(2.44-4.69) | -0.8(-1.09--0.51) | |
| Andean Latin America | | 1.25(0.92-1.68) | |  | | 6.1(4.45-8.19) |  | | | 3.13(2.18-4.27) | |  | | 5.68(3.93-7.71) | -0.28(-0.38--0.18) | |
| Central Latin America | | 6.04(4.58-7.69) | |  | | 6.96(5.28-8.81) |  | | | 14.76(11-18.94) | |  | | 6.24(4.65-8.02) | -0.53(-0.63--0.44) | |
| Tropical Latin America | | 2.07(1.55-2.72) | |  | | 2.25(1.69-2.96) |  | | | 4.83(3.62-6.21) | |  | | 2(1.5-2.56) | -0.29(-0.51--0.08) | |
| North Africa and Middle East | | 6.02(4.27-8.13) | |  | | 4.24(2.92-5.86) |  | | | 14.48(10.13-20.2) | |  | | 3.84(2.71-5.3) | -0.12(-0.26-0.01) | |
| South Asia | | 11.57(8.58-15.3) | |  | | 2.01(1.51-2.62) |  | | | 21.9(16.59-28.26) | |  | | 1.6(1.21-2.05) | -1.2(-1.4--1) | |
| Central Sub-Saharan Africa | | 0.74(0.5-1.05) | |  | | 3.32(2.3-4.7) |  | | | 1.55(0.99-2.32) | |  | | 2.89(1.9-4.24) | -0.75(-0.87--0.64) | |
| Eastern Sub-Saharan Africa | | 3.54(2.45-4.93) | |  | | 5.02(3.53-6.94) |  | | | 6.73(4.78-9.13) | |  | | 4.4(3.14-5.94) | -0.56(-0.62--0.51) | |
| Southern Sub-Saharan Africa | | 0.7(0.5-0.94) | |  | | 2.51(1.72-3.41) |  | | | 1.32(1.04-1.67) | |  | | 2.41(1.91-3.01) | -0.33(-0.75-0.09) | |
| Western Sub-Saharan Africa | | 3.73(2.55-5.47) | |  | | 4.49(3.06-6.53) |  | | | 6.87(4.81-9.46) | |  | | 3.8(2.68-5.19) | -0.58(-0.63--0.52) | |
| Note: ASDR, age-standardized death rate; SDI, social-demographic index; EAPC, estimated annual percentage change. | | | | | | | | | | | | | | | | |

| **Table S4.** The DALYs of NAFLD in 1990/2019 and temporal trends | | | | | | | |  |
| --- | --- | --- | --- | --- | --- | --- | --- | --- |
| Characteristics | 1990 | | |  | 2019 | | | 1990-2019 |
|  | DALYs No×10^3^ (95% CI) |  | Age standardized DALYs/10^5^ No. (95% CI) |  | DALYs No×10^3^ (95% CI) |  | Age standardized DALYs/10^5^ No. (95% CI) | EAPC No. (95% CI) |
| Overall | 2711.27(2078.58-3478.94) |  | 63.28(48.58-80.86) |  | 4417.28(3348.22-5671.2) |  | 53.33(40.73-68.29) | -0.82(-0.93--0.7) |
| Sex |  |  |  |  |  |  |  |  |
| Male | 1505.98(1104.46-1972.29) |  | 72.2(54.06-93.52) |  | 2528.55(1894.89-3311.25) |  | 54.51(42.12-69.54) | -0.72(-0.83--0.6) |
| Female | 1205.29(932.77-1536.54) |  | 62.98(47.7-81.89) |  | 1888.73(1463.88-2375.66) |  | 43.92(34.03-55.32) | -0.94(-1.05--0.83) |
| Socio-demographic factor |  |  |  |  |  |  |  |  |
| High SDI | 365.87(275.87-477.98) |  | 37.26(27.74-48.87) |  | 554.91(423.15-707.93) |  | 34.16(26.14-44.21) | -0.42(-0.5--0.35) |
| High-middle SDI | 575.74(442.31-725.9) |  | 52.09(40.11-65.58) |  | 813.58(613.15-1052.67) |  | 41.67(31.71-53.6) | -0.95(-1.07--0.83) |
| Middle SDI | 1002.51(773.83-1265.3) |  | 86.1(67.01-107.57) |  | 1679.78(1270.33-2139.73) |  | 65.4(50.66-82.14) | -1.17(-1.33--1.02) |
| Low-middle SDI | 526.68(389.4-703.67) |  | 73.8(55.05-95.91) |  | 948.33(698.97-1251.28) |  | 63.25(47.35-82.85) | -0.81(-1.02--0.6) |
| Low SDI | 238.59(171.42-331.23) |  | 86.65(62.74-117.91) |  | 417.42(300.27-562.04) |  | 68.06(49.42-91.9) | -1.02(-1.09--0.95) |
| Region |  |  |  |  |  |  |  |  |
| East Asia | 637.9(500.55-806.51) |  | 65.25(51.77-81.38) |  | 630.14(481.51-782.01) |  | 30.28(23.4-37.39) | -3.42(-3.82--3.03) |
| Southeast Asia | 334.44(244.6-444.27) |  | 109.76(81.15-144.49) |  | 635.25(464.29-836.79) |  | 98.16(73.28-126.92) | -0.37(-0.43--0.31) |
| Oceania | 1.68(1.17-2.41) |  | 42.59(30.36-59.09) |  | 3.73(2.44-5.49) |  | 40.15(27.11-56.22) | -0.15(-0.18--0.11) |
| Central Asia | 28.16(20.33-38.08) |  | 55.32(39.54-74.56) |  | 95.21(67.8-131.25) |  | 112.39(81.58-151.17) | 2.30(1.92-2.69) |
| Central Europe | 58.5(43.04-77.61) |  | 40.28(30.09-52.96) |  | 66.35(47.15-91.42) |  | 36.13(25.46-49.93) | -0.51(-0.67--0.35) |
| Eastern Europe | 83.52(60.1-113.55) |  | 30.49(22.13-40.6) |  | 263.01(183.27-361.83) |  | 91.22(63.75-127.09) | 4.06(3.28-4.85) |
| High-income Asia Pacific | 57.39(46.39-70.46) |  | 27.97(22.63-34.2) |  | 68.82(55.49-82.92) |  | 17.25(13.94-20.86) | -2.15(-2.48--1.81) |
| Australasia | 6.73(4.95-8.9) |  | 29.9(21.92-40.29) |  | 13.91(10.82-17.36) |  | 32.08(24.94-40.72) | 0.65(0.4-0.91) |
| Western Europe | 254.63(184.16-340.56) |  | 49.07(35.48-65.56) |  | 250(188.02-320.23) |  | 32.8(24.61-42.87) | -1.62(-1.71--1.53) |
| Southern Latin America | 25.79(17.74-36.26) |  | 55.24(38.08-77.69) |  | 38.19(26.91-52.64) |  | 47.56(33.39-65.99) | -0.26(-0.48--0.05) |
| High-income North America | 115.89(86.66-152.26) |  | 35.83(26.33-47.46) |  | 236.66(178.74-311.2) |  | 43.04(32.54-56.6) | 0.82(0.6-1.05) |
| Caribbean | 28.56(21-38.28) |  | 104.15(76.67-138.37) |  | 45.1(30.71-63.86) |  | 87.48(59.86-123.71) | -0.82(-1.12--0.52) |
| Andean Latin America | 35.41(25.41-48.45) |  | 154(110.25-209.78) |  | 73.96(50.8-102.84) |  | 128.67(88.56-178.85) | -0.73(-0.85--0.61) |
| Central Latin America | 187.58(142.3-243.49) |  | 188.76(141.59-244.26) |  | 396.84(291-522.91) |  | 161.39(118.97-212.38) | -0.77(-0.9--0.64) |
| Tropical Latin America | 65.39(47.81-87.46) |  | 60.38(44.4-79.55) |  | 128.79(93.98-169.79) |  | 51.56(37.88-67.46) | -0.47(-0.66--0.28) |
| North Africa and Middle East | 142.84(104.85-189.17) |  | 82.7(59.85-110.82) |  | 336.02(235.32-461.22) |  | 76.39(53.56-104.61) | -0.06(-0.19-0.07) |
| South Asia | 392.53(285.99-525.73) |  | 54.98(40.85-72.78) |  | 651.07(487.49-865.51) |  | 41.62(31.43-54.69) | -1.42(-1.7--1.13) |
| Central Sub-Saharan Africa | 23.68(15.95-34.69) |  | 85.74(58.63-122.02) |  | 50.35(32.15-77.03) |  | 74.24(47.51-110.75) | -0.75(-0.86--0.64) |
| Eastern Sub-Saharan Africa | 102.55(69.63-144.1) |  | 120.23(83.43-167.08) |  | 192.73(134.39-268.18) |  | 101.32(71.94-139.19) | -0.72(-0.78--0.67) |
| Southern Sub-Saharan Africa | 21.65(15.54-29.57) |  | 66.17(46.96-90.54) |  | 38.06(29.49-48.86) |  | 59.97(47.1-75.87) | -0.50(-0.94--0.05) |
| Western Sub-Saharan Africa | 106.43(71.42-156.12) |  | 107.51(72.73-159.03) |  | 203.09(137.26-292.86) |  | 89.75(62.2-124.88) | -0.62(-0.67--0.58) |
| Note: DALYs, disability-adjusted life-years; SDI, social-demographic index; EAPC, estimated annual percentage change. | | | | | | | | |
