# Supplementary figures and images for: The Global, Regional, and National Burden and Trends of NAFLD in 204 Countries and Territories: An Analysis From Global Burden of Disease 2019
Source: JMIR Public Health Surveill. 2022 Dec 12;8(12):e34809. doi: 10.2196/34809 (PMC9793331; doi:10.2196/34809)

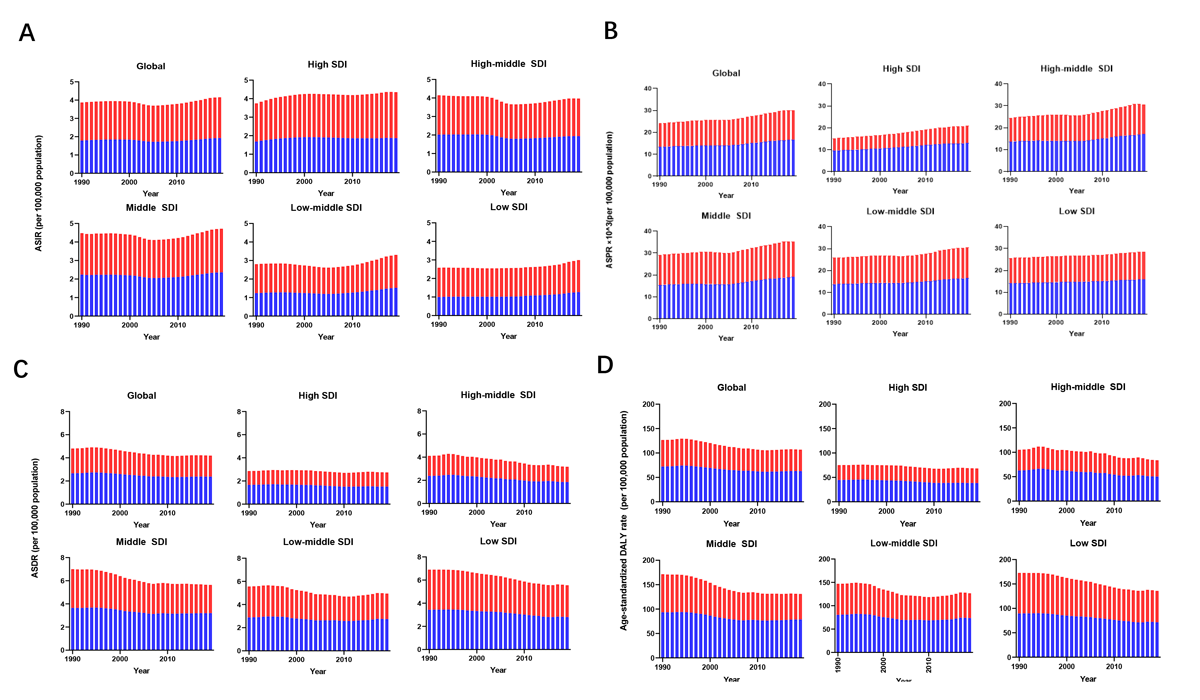

Supplement: Multimedia Appendix 3 [file publichealth_v8i12e34809_app3.png]
